# Imaging active faulting in the western Taiwan Strait

**DOI:** 10.1038/s41598-020-60666-3

**Published:** 2020-02-28

**Authors:** Yifeng Zhang, Hao Kuo-Chen, Joaquina Alvarez-Marron, Dennis Brown, Andrew Tien-Shun Lin, Zhizhao Xie, Xing Jin

**Affiliations:** 1 Institute of Marine Earthquake, China Earthquake Administration, Xiamen, China; 2 Earthquake Administration of Fujian Province, Fuzhou, China; 3 Department of Earth Sciences, National Central University, Jhongli, Taiwan; 4 Institute of Earth Sciences, Jaume Almera, ICTJA-CSIC, 08028 Barcelona, Spain

**Keywords:** Seismology, Tectonics

## Abstract

Large, destructive historical earthquakes off the coast of China’s Fujian Province point to important tectonic activity in the western Taiwan Strait that, until recently, has received little attention. We present newly acquired reflection seismic data that is used to study the shallow crustal structure of the western Taiwan Strait. With these data we map the location of the Benhai fault for the first time, describe its upper crustal geometry and, in combination with seismicity and earthquake focal mechanisms, interpret its kinematics. These new data demonstrate that there is wide spread evidence of faulting that reaches the sea floor in the western Taiwan Strait, clearly indicating that the Benhai fault is active. Faults that cut up section from steep basin sidewalls to form flower structures or terraced sidewall fault zones, together with the fault and basin map pattern, are consistent with this fault zone being in the early developmental stages of a dextral strike-slip system. Earthquake focal mechanisms, although not definitive, support the model of an active dextral strike-slip fault system in the western Taiwan Strait.

## Introduction

**Figure 1:**
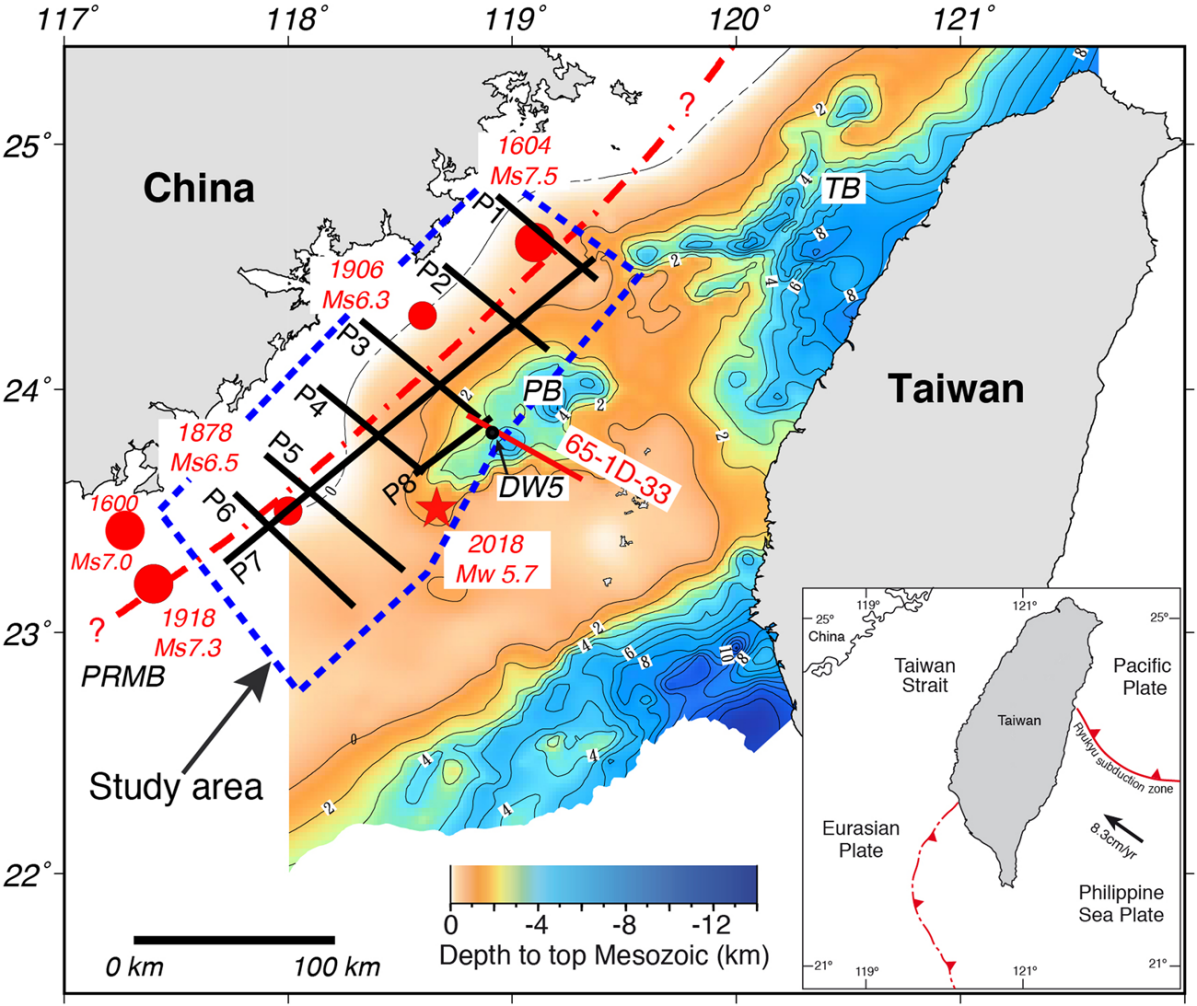
Map of the Taiwan Strait showing the locations of the study area, the reflection seismic profiles (P1 through P8) used in the study, and large historical earthquake (red circles) magnitudes and locations are from ref. ^36^. The red star is the Mw 5.7 earthquake (USGS) that occurred in 2018. The depth to the top of the Mesozoic basement map in the strait is from ref. ^3^. The red line is the reflection seismic profile (65-1D-33) from Chinese Petroleum Corporation of ref. ^2^. The dashed red line is the proposed location of the Benhai fault that has been suggested by previous workers^6–8^. TB = Taihsi Basin, PB = Penghu Basin, PRMB = Pearl River Mouth Basin. M_S_ = surface-wave magnitude, M_W_ = moment magnitude. DW5 is the borehole shown in Fig. 2. The inset shows the plate tectonic setting of the Taiwan arc-continent collision.

Despite a number of large, destructive historical earthquakes having occurred in the western Taiwan Strait (Fig. 1) (see Methodology for the data base of historical events used in this study), little is known about its geological structure or the potential risk that this area could pose for people and infrastructure along the coast of China’s Fujian Province. In large part, this paucity of knowledge is because little geoscientific attention has focused on the Taiwan Strait since the 1970’s and 1980’s when extensive reflection seismic and drilling campaigns^1–3^ failed to identify significant petroleum potential. This lack of attention was further compounded by the fact that much of the data collected during this time has not been made available to the general scientific community for more detailed studies, and undertaking new studies has been complicated by territorial disputes. This meant that until recently^4,5^, little scientific research was carried out there. Nevertheless, based on historical and recent seismicity, sparse shallow reflection seismic data, refs. ^6–8^ have suggested that there is a roughly NE-striking fault system, called the Binhai fault, that skirts the coast of China from the east of the island of Hainan, along the western margin of the Pearl River Basin, and through the western Taiwan Strait into the East China Sea Basin (Fig. 1). Yet, the existence of this fault and its possible location, geometry, and kinematics are still not certain.

The part of the continental margin that comprises the Taiwan Strait consists of a broad shelf area with a number of deep, fault-bounded basins^9–15^. The extensional tectonic history of this area began with rifting during the Early to Late Eocene and, by the late Early Oligocene, had evolved to sea-floor spreading and eventually the formation of the South China Sea ocean basin to the south^2,14^. The late Early Oligocene change from rifting to sea-floor spreading in the South China Sea is recorded in the stratigraphy of the Taiwan Strait by what is commonly called the break-up unconformity^2,3,14^. Further extension during the Middle and Late Miocene affected much of the margin to various degrees, but was particularly felt on the outer part of the shelf, facing the South China Sea^2^. On the shelf, the Miocene extension was accompanied by the eruption of extensive intraplate flood basalts, forming the Penghu Archipelago^16–18^. Since the Late Miocene onset of arc-continent collision in Taiwan, a westward advancing foreland basin has been forming in the strait, and its sediments now reach to approximately 120° E^1,19^.

Far-field stress from the Taiwan arc-continent collision is causing reactivation of Cenozoic faults and widespread, but moderate, low magnitiude seismicity in the eastern and central part of the Taiwan Strait^20–22^. Yet, much of the western part of the strait is thought to be an area of relative tectonic quiescence despite widespread evidence for active faulting to the south, in the Pearl River Mouth Basin^7,8,23–26^ and to the north, in the Taibei Depression^27,28^. Ref. ^28^ shows a marked crustal-scale P-wave velocity anomaly just off the southeast coast of Fujian that may indicate continuance of faulting from the Pearl River Mouth Basin through the western Taiwan Strait, but this has yet to be tested. In this study, we use newly acquired reflection seismic data in the western Taiwan Strait (Fig. 1) to accurately map the location of the Benhai fault for the first time, to ascertain its upper crustal geometry and, in combination with seismicity and earthquake focal mechanisms, to determine its kinematics.

## Results

### Reflection seismic data

**Figure 2:**
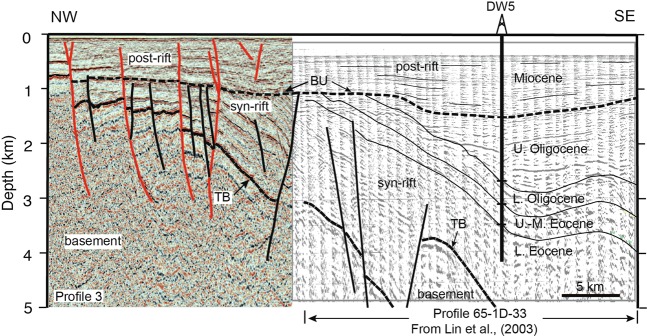
Stratigraphic correlation between the eastern part of profile 3 the western part of profile 65-1D-33 of ref. ^3^. The important features to note are the top of the basement (TB) and the break-up unconformity (BU). Stratigraphic ages are derived from borehole DW5 whose location is shown in Fig. 1. Sediments below the break-up unconformity are termed syn-rift and those above it are termed post-rift.

**Figure 3:**
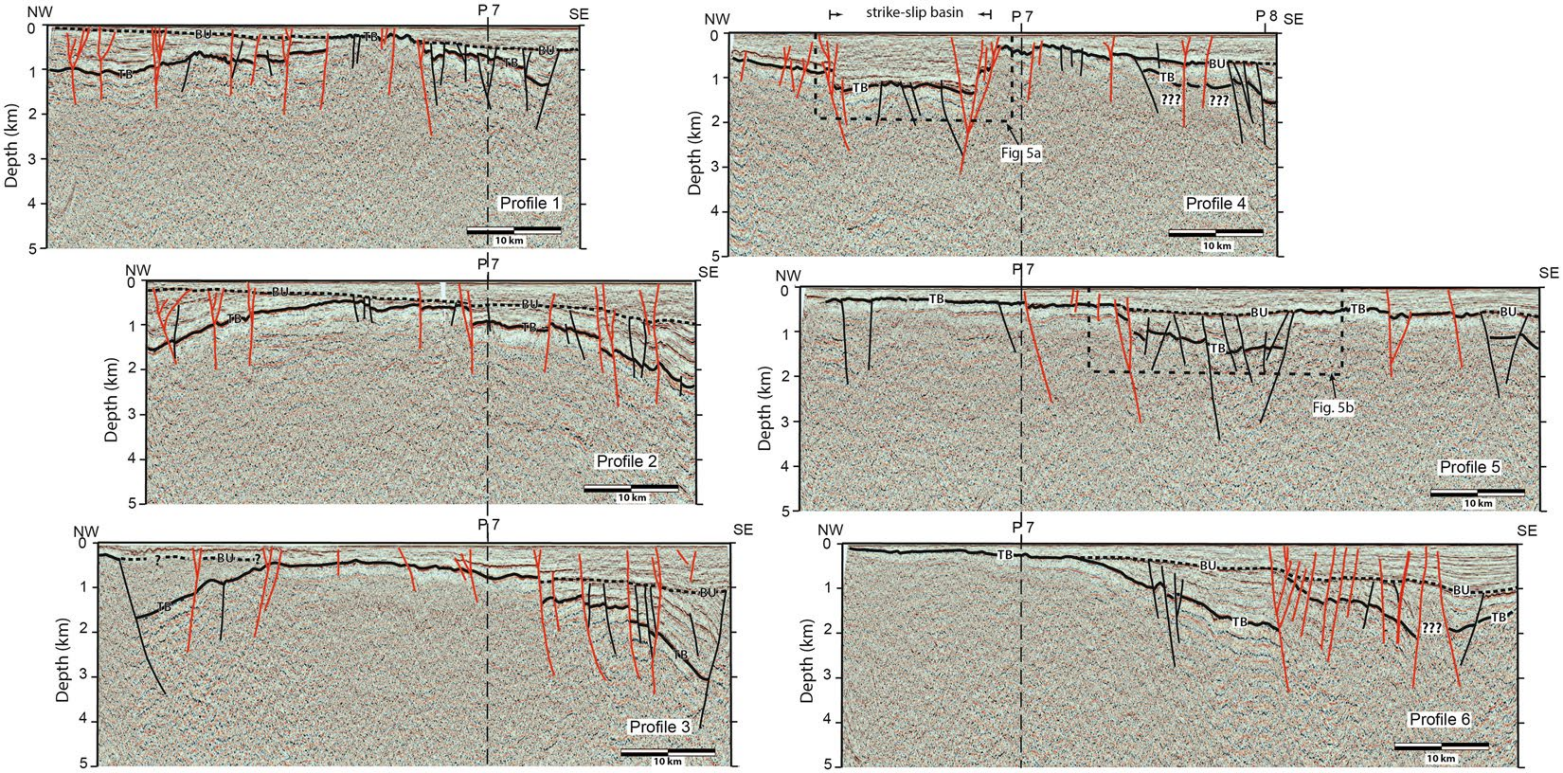
Interpretations of profiles 1 through 6. The dashed black line labelled BU is the break-up unconformity and the solid black line labelled TB is the top of the basement. Black faults do not cut the break-up unconformity, whereas red faults do. The locations of crossing profiles 7 and 8 are shown, as are the locations of Fig. 5A,B.

**Figure 4:**
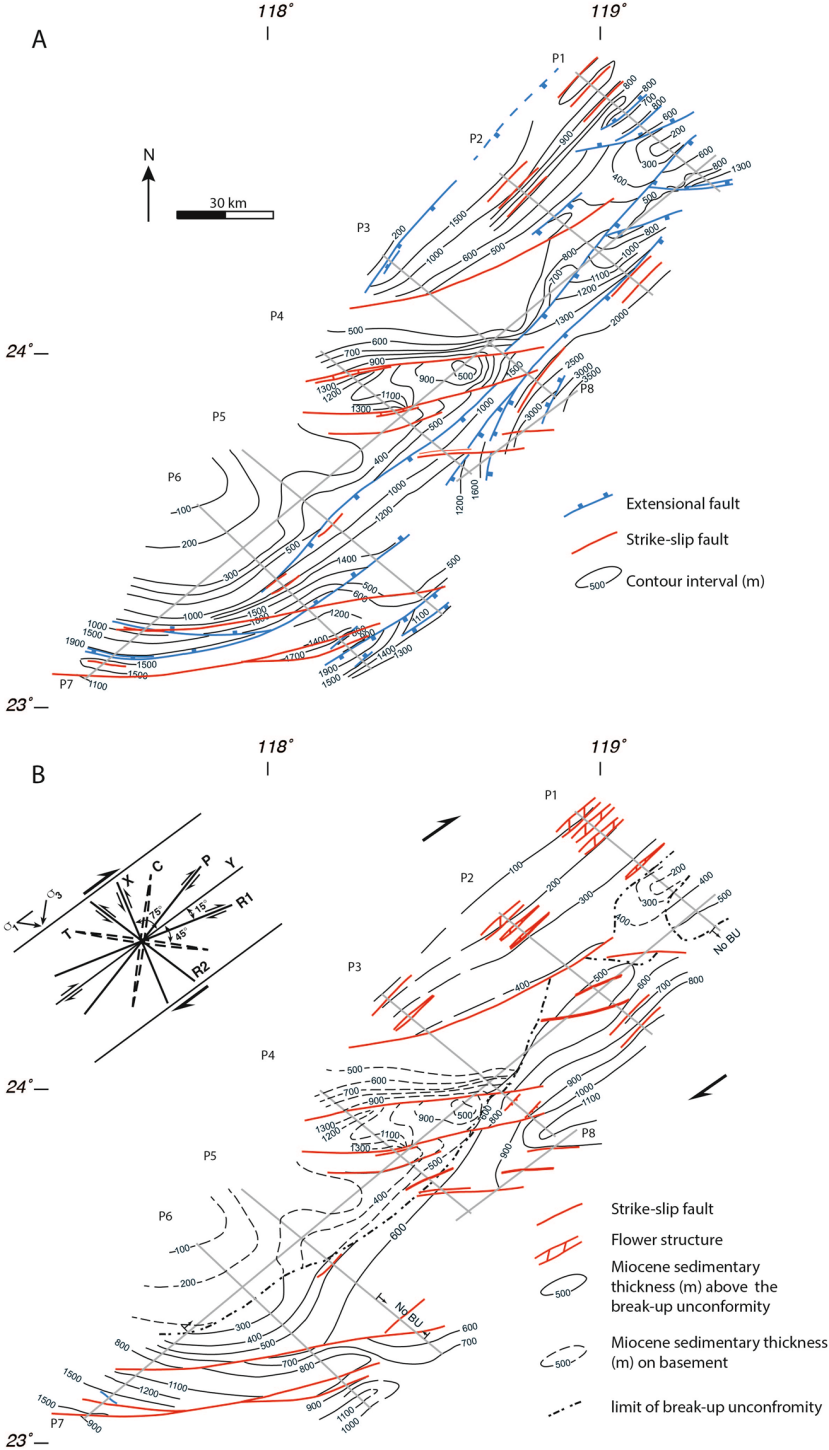
Depth contour maps to the top of the basement (**A**) and to the break-up unconformity (**B**) interpreted from the seismic profiles (shown as gray lines and labelled P1 through P8). The extensional faults in (blue) are Eocene-aged syn-rift faults, whereas the strike-slip faults (red) are post break-up unconformity and can be considered as being active. The map pattern of the strike-slip faults suggests that it is a northeast striking, dextral system (see the inset).

The age of the various stratigraphic units and their contacts have been correlated between our profile 3 and profile 65-1D-33 of ref. ^2^, with constraints provided by borehole DW5 located in the Penghu Basin (Figs. 1 and 2). Two surfaces are of particular importance: (1) the top of the basement and, (2) the break-up unconformity (TB and BU, respectively in Fig. 2). According to ref. ^2^, the ages of TB and BU are ~66–58 Ma and ~37–30 Ma, respectively, which separate three episodes (pre-rift, syn-rift, and post-rift) of the rifting processes related to the opening of the South China Sea. Because these two unconformities are the most pronounced unconformities shown in all the seismic profiles (Fig. 3), we are able to use them to identify the cross-cutting relationship between the unconformities and the faults. Based on this, faults that do not cut the break-up unconformity (shown in black) range from Eocene to Early Oligocene in age, whereas those that do cut it (shown in red) are younger. If a fault reaches the sea floor, it is currently active (although faults that do not breach the surface may also be seismically active). Below, we present the interpretation of profiles 1 through 6 (Fig. 3). The un-interpreted versions are presented in Supplementary Figure S1. Profiles 7 and 8 provided tie profiles that helped with the correlation of the structure between profiles 1 through 6. Interpreted versions of these are given in Supplementary Figure S2. To further aid with the structural interpretation, fault maps with depth contours of the top of the basement and the break-up unconformity reflections have been constructed (Fig. 4).

**Figure 5:**
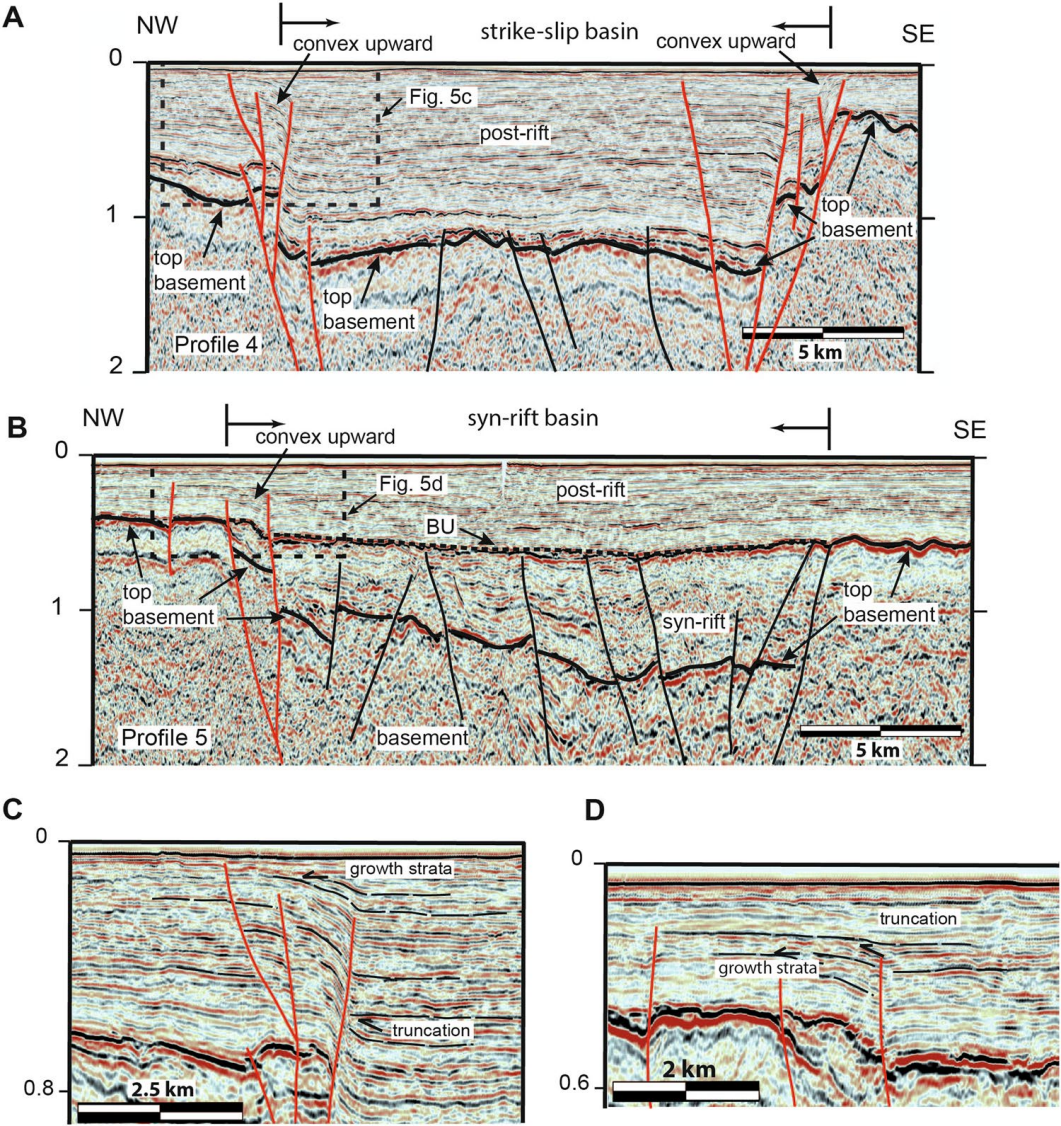
Detailed interpretations showing (**A**) strike-slip faults that cut the break-up unconformity, reaching almost to the surface and, (**B**) extensional faults that do not cut the break-up unconformity. In the west (**C**), the reactivation of one basin bounding fault has caused it to cut the break-up unconformity and concave upward reflections is suggestive of a strike-slip sense of movement. Growth strata and truncation of reflections (**D**) indicate that faults are active well into the post-rift sedimentary sequence. The locations of Figures A and B are shown in Fig. 3, whereas that of C and D are shown in A and B.

In all profiles, the top of the basement (TB in Fig. 3) coincides with a high-amplitude reflection below which the crust is overall acoustically transparent. Only locally is there sufficient reflectivity to allow minimal interpretation of the basement structure. In profiles 1, 2, and 3, the top of the basement has an overall domed shape, and it is cut by numerous faults. A northeast-striking basement high extends from profile 4 to profile 1 and, locally, the post-rift sequences unconformably overlie the basement (Figs. 3 and 4A). In both the west and the east, the top of the basement reflection reaches to between 1 km and 3 km depth. Up to 2 km thick packages of inclined reflections with, commonly, a fan-like geometry, correlate with the Eocene and Early Oligocene syn-rift sediments. Reflections that are attributed to the syn-rift sediments generally terminate against the basement along the steeply inclined basin bounding faults. In profiles 4, 5, and 6, the top of the basement reflector reaches much shallower depths, being less than a hundred meters deep in the west (e.g., profile 6). In profiles 5 and 6, syn-rift basins are filled with up to a kilometer of sediments (Fig. 5). In profile 6, the top of the basement reflector deepens toward the east, where it is offset by several faults with asymmetric packages of syn-rift sediments forming distinct basins.

The seismic characteristics related to the syn-rift sediments from the tie line of Fig. 2 are also observed in profiles 1, 2, and 3. The syn-rift sediments were truncated at the top by a high-amplitude, gently eastward dipping reflection that can be correlated with the regional break-up unconformity (BU in Fig. 3). Throughout the study area, the break-up unconformity reflection often terminates against basement highs that are generally, but not always (see profile 6), fault bounded. The reflection package above the break-up unconformity is related to the Miocene and younger post-rift sediments^2^ (Fig. 3). The thickness of this package of reflections increases from around 100 meters in the west to more than 1000 meters in the east.

Throughout the study area, a number of faults cut both the top of the basement and the break-up unconformity, and extend to the surface. In many cases, such as in the western part of profiles 1, 2, and 3, and the basin in profile 4, these faults often display convex upward or concave downward warping of reflections that are crosscut by a system of faults that merge at depth (Fig. 5A,B). The geometry of these reflections and the associated fault systems is suggestive of positive and negative strike-slip flower structures. In profile 4, a roughly one km deep fault-bounded basin with nearly horizontal reflections within the basin sedimentary sequence is suggestive of a strike-slip basin. The basin bounding faults cut both the top of the basement and the break-up unconformity reflections (Fig. 5A) and can be traced east-northeast through profile 7 (Supplementary data Figure S2) onto profile 3 where they are weakly developed (Fig. 4). Throughout the study area, there is widespread evidence of growth strata and/or truncation of reflections above the break-up unconformity (Fig. 5C,D), providing evidence for syn-tectonic sedimentation from the Miocene to the Present.

Faults that cut the top of the basement, but not the break-up unconformity are widespread in the study area (Figs. 4 and 5), although they are mostly found in the eastern part of the profiles (1, 2, 3, and 4), where they image the western flank of the Penghu basin. A roughly 15 km wide and 1 km deep basin imaged in profile 5 provides a particularly good example of a syn-rift, fault bounded, basin (Fig. 5B). We interpret this basin to strike northeast, appearing in the eastern end of profile 4 and also in profile 6 (Fig. 4). In all three profiles, there are also faults that clearly cut through the break-up unconformity. Several of these reach the surface indicating that they are currently active. In profile 4, the western bounding fault of the basin also offsets the break-up unconformity, extending upward into the post-rift sequences, suggesting it has been reactivated (Fig. 5A).

### Seismicity data

**Figure 6:**
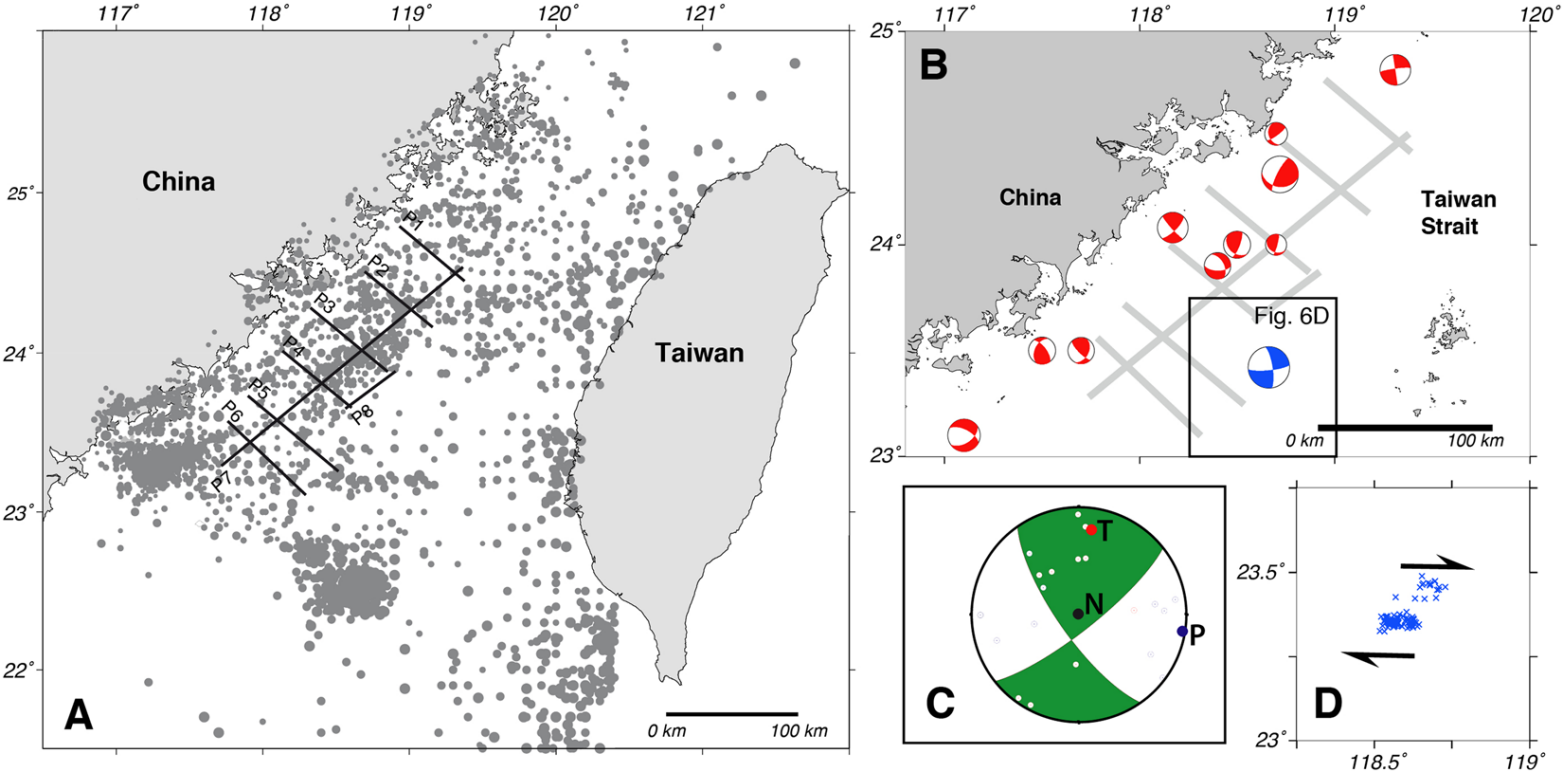
(**A**) Recent seismicity in the Taiwan Strait (up to M_S_ 5.2). For the sake of clarity, all seismicity has been removed from on land Taiwan and China. (**B**) Focal mechanisms in red color coded determined by EAFP. Focal mechanism of the Mw 5.7 earthquake in blue color coded determined by USGS (https://earthquake.usgs.gov/earthquakes/eventpage/us1000hwix/moment-tensor, last accessed on January 2019). (**C**) Composite focal mechanisms of 10 events. P-axis (blue circle): 98.8° and T-axis (red circle): 7.8°. The black dot (N) is the null axis. Black dots within circles are the P and T axes of the 10 events used in the determination of the composite focal mechanism. The composite focal mechanism was determined using the FaultKin software of ref. ^37^. (**D**) Aftershock sequence of the Mw 5.7 earthquake in 2018. Blue crosshair: aftershock.

**Figure 7:**
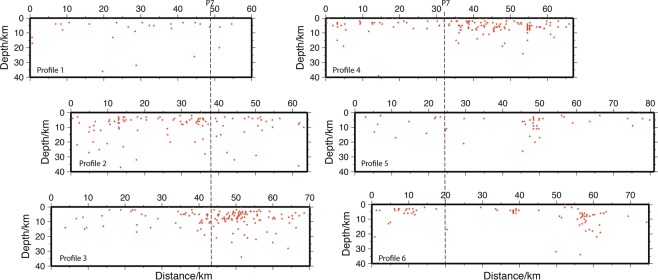
Seismicity along each of Profiles 1 through 6. Events have been projected 15 km from either side of the profiles.

Seismicity is predominantly clustered in the central part of the study area, with two well-defined clusters to the southeast and southwest (Fig. 6A). Elsewhere, seismicity is evenly scattered throughout. Focal mechanisms were determined for 10 events of between Ms 3 and 5.2 (Ms is the magnitude determined from surface waves) using P-wave first motion polarity. These show a range of fault types, from oblique extension and thrusting through to strike-slip (Fig. 6B), indicative of the complex fault types commonly found in strike-slip fault systems. It is interesting to note, however, that a composite focal mechanism for 10 events gives a strike-slip fault sense with a P-axis that plunges gently toward 98.8° (Fig. 6C), sub-parallel to the relative motion vector between the Philippine Sea and Eurasian plates (306°) (Fig. 1). Throughout the study area, the majority of seismicity has been in the upper 10 km of the crust, with some events reaching as deep as 30 km (Fig. 7). Along profiles 1 and 2, seismicity is mostly diffuse, with weak clustering between kms 10 to 20 in profile 2 where faults are interpreted to breach the break-up unconformity, and from kms 30 to the end of the profile where a number of faults are interpreted to reach the surface. In profiles 3 and 4, there is clustering of seismicity from c. km 35 to the eastern end of the profiles. This clustering coincides with the western flank of the Penghu Basin where numerous faults are interpreted to reach the surface. There is very little seismicity associated with the area beneath the basin imaged in the central part of profile 4. Only a small number of events were recorded beneath the basin imaged in the west of profiles 1, 2, and 3, although there is a small cluster between 0 and 20 km in profile 2. The western part of profile 5 displays sparse events, although there is a small cluster at around km 10 in profile 6. Eastward, both profiles 5 and 6 have clustering of events between km 50 and km 60 that coincide with active faults interpreted in this area.

In 2018, a series of earthquakes occurred in the Taiwan Strait, with the Mw 5.7 (USGS) main shock being near the study area (Fig. 1). We have relocated (see Methodology for how the relocation was carried out) the main shock and aftershocks by jointly using the data from both sides of the Taiwan Strait in order to have a better constraint on the event locations. In total, we relocated 101 events with depths that range from 5 to 20 km. Based on the focal mechanism from the USGS and the distribution of the aftershocks, the sesimogenic zone is a dextral fault system with the P-axis plunging gently toward 308°, parallel to the relative motion vector between the Eurasia and the Philippine Sea plates (Fig. 6D).

## Discussion

The western Taiwan Strait has been typically shown on maps as tectonically quiescent, with often little more interpretation than the presence of rift-related Paleogene basins^3,6,24,29^ or, at the most, with strike-slip faults on land along the coast of Fujian Province^2,4,30^. Nevertheless, several seismicity studies present data that indicate that this area is seismically active^4,6,8^ although, to date, no reflection seismic data has provided unequivocal images of active faults. The historical large earthquakes (Fig. 1) likely have large uncertainties since they occurred before the use of modern seismometers, but their locations coincide with those of the background seismicity (Fig. 6A) and the many active faults shown in the seismic profiles (Fig. 3). It is reasonable to infer that the seismogenic zones in this region are highly active and are related to those large earthquakes that occurred during the period 1600–1918. Our new reflection seismic profiles provide important new data that allows us to place constraints on the location and structural style of the Benhai fault system in the western Taiwan Strait. It shows that, unlike the discrete fault interpreted by previous authors^6–8^ (Fig. 1), the Benhai fault system is a more than 75 km wide diffuse zone of faulting. Seismicity suggests that certain parts of the fault system are active to depths of greater than 20 km, but that the majority of activity occurs in the upper 10 km of the crust (Fig. 7).

Our data clearly shows a number of faults that cut the break-up unconformity and, in many cases, reach the sea floor. In several cases, these active faults cut up section from steep basin sidewalls to form flower structures or terraced sidewall fault zones (Fig. 5), forming structures that are similar to those described from strike-slip pull-apart basins^31,32^. Furthermore, the fault and basin map pattern (Fig. 4) is also consistent with that expected for the early stages of development of a dextral strike-slip system^32^. Based on the comparison of our reflection seismic data interpretation with the analog models of ref. ^32^ and with reflection seismic profiles in areas of strike-slip faulting^33–35^, we interpret the structure of the Binhai fault in the western Taiwan Strait to be that of the dextral strike-slip system. Furthermore, in a number of cases (e.g., profiles 3 and 7; Fig. 3) these active strike-slip faults at depth form basin-bounding faults to syn-rift basin, providing strong evidence that, at least locally, they are reactivating pre-existing faults. Finally, growth strata and truncation reflection geometries high up within the post-rift sedimentary packages (Fig. 5C,D) suggests that the Binhai fault system has been active since the Miocene.

## Methodology

### Reflection seismic data

**Figure 8:**
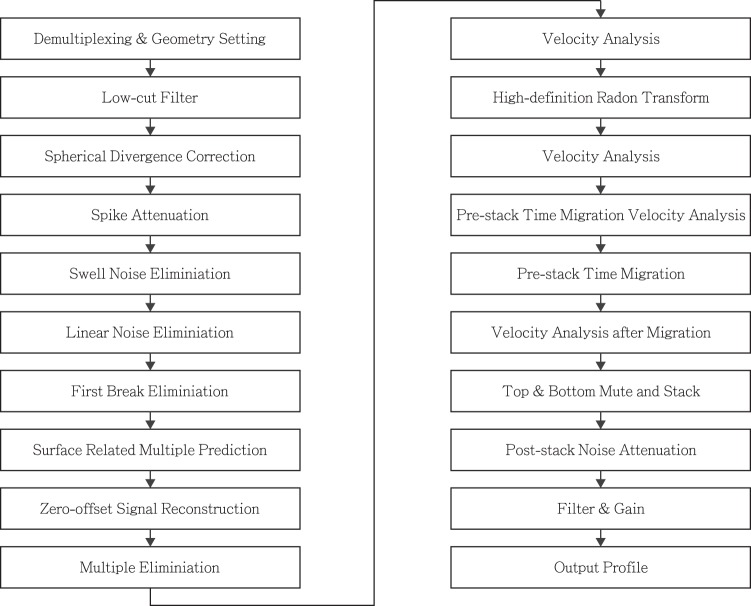
The detailed processing flow of the data.

In May 2017, eight multichannel seismic reflection (MCS) profiles were acquired in the western Taiwan Strait with the express purpose of locating and imaging the active Benhai fault system (Fig. 1). A GGUN array, comprised of 2 air guns with 520 CI and 2 with 150 CI was used, providing a total compressive pressure of 2000 PSI. A 1350 meter-long Sentinel streamer containing 108 hydrophones spaced at 12.5 m was towed at 8 meters depth below sea level. The shooting interval was every 37.5m. The CDP (Common Depth Point) interval and the maximum fold are 6.25m and 18, respectively. All profiles were recorded for 3.5 seconds, except profile 5 which was recorded for 3.2 seconds. The Seal 408XL recording system with a standard SEGD output format was used. The sampling rate was 1ms. A 3 Hz to 200Hz bandpass filter was applied for the data. Navseis with NAVCOM SF-3050 GPS was used for the navigation system. The total survey length of these MCS profiles is 700km. The detailed processing flow of the data is shown in Fig. 8.

### Seismicity data

Since 1971 the Earthquake Administration of Fujian Province (EAFP) has deployed seismic stations throughout Fujian Province, including 97 broad-band stations deployed between 2001 through 2014. During this time, numerous earthquakes of up to M_S_ 5.2 (M_S_ is the magnitude determined using surface waves; a commonly used method in China) were recorded in the study area of the western Taiwan Strait (Fig. 1). This is the data base used in this study. These hypocenters have all been located using the Hypo71 travel-time methodology in the 1D S-wave velocity model of ref. ^5^. Events were then projected onto the sections corresponding to the reflection seismic data from a maximum of 15 km either side (Fig. 7). The spatial uncertainty of the hypocenter locations is about 5 km in the horizontal and vertical.

The historical earthquake magnitudes and locations shown in Fig. 1 are from ref. ^36^.

## Supplementary information

Dataset 1 and dataset 2.
