# Supplementary material for: Imaging active faulting in the western Taiwan Strait
Source: Sci Rep. 2020 Feb 28;10:3703. doi: 10.1038/s41598-020-60666-3 (PMC7048765; doi:10.1038/s41598-020-60666-3)
Supplement: Supplementary file 1 — Dataset 1 and dataset 2. [file 41598_2020_60666_MOESM1_ESM.pdf]

Supplement Information to

Imaging active faulting in the western Taiwan Strait

By Yifeng Zhan, Hao Kuo-Chen, Joaquina Alvarez-Marron, Dennis Brown, Andrew Lin, Zhizhao Xie, Xing Jin

These two supplementary figures are uninterpreted profiles 1-6 (Figure S1) and interpreted profiles 7 and 8 (Figure S2)

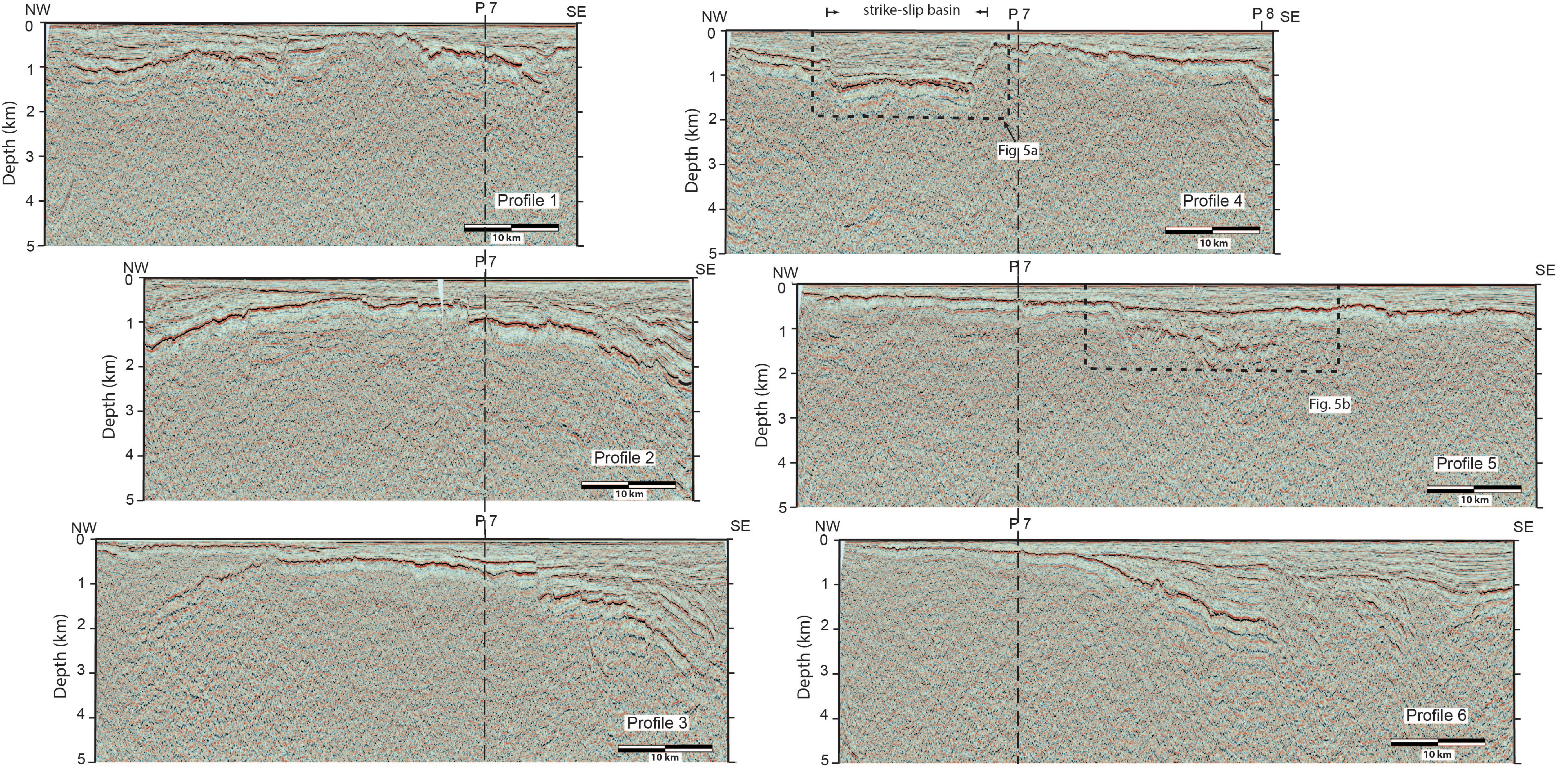

NW
P 7
SE
Depth (km)
0
1
2
3
4
5
Profile 1
10 km
strike-slip basin
P 8
Fig. 5a
Profile 4
Profile 2
Fig. 5b
Profile 5
Profile 3
Profile 6

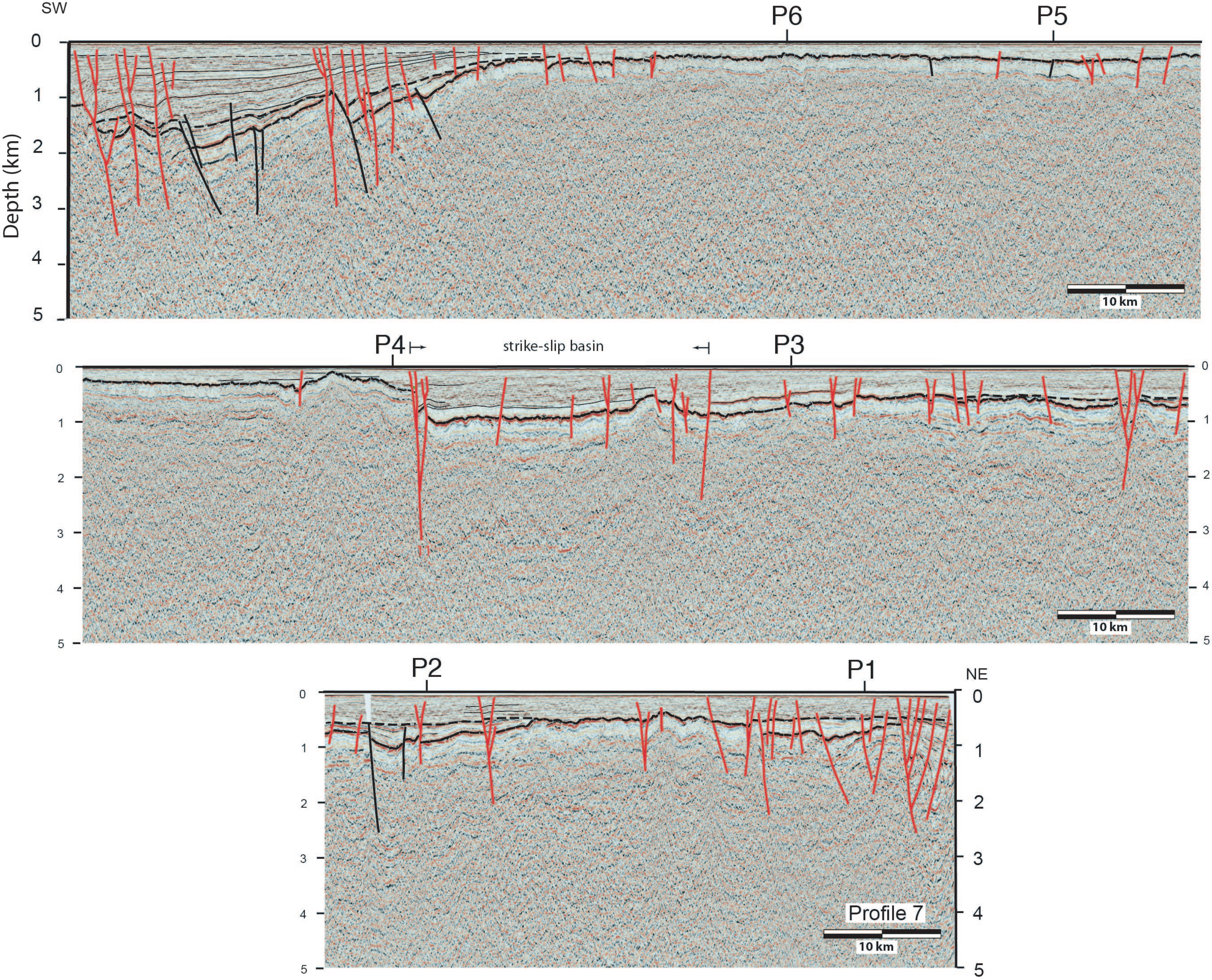

SW
P6
P5
Depth (km)
10 km
P4
strike-slip basin
P3
P2
P1
NE
Profile 7

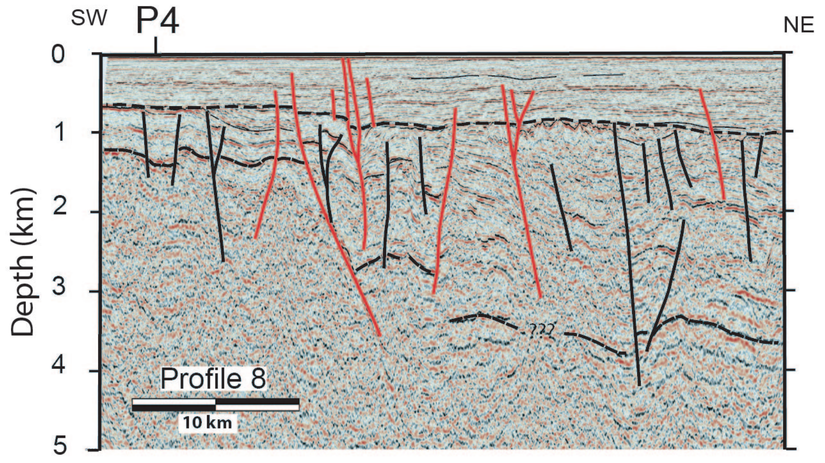

SW
P4
NE
Depth (km)
Profile 8
10 km
